# Research of activity of Main Belt Comets 176P/LINEAR, 238P/Read and 288P/(300163) 2006 VW_139_

**DOI:** 10.1038/s41598-019-41880-0

**Published:** 2019-04-02

**Authors:** Jianchun Shi, Yuehua Ma, He Liang, Ruiqi Xu

**Affiliations:** 1 Purple Mountain Observatory, Chinese Academy of Sciences, Nanjing, 210034 China; 2 CAS Key Laboratory of Planetary Sciences, Chinese Academy of Sciences, Nanjing, 210034 China; 3 CAS Center for Excellence in Comparative Planetology, Hefei, China; 4 School of Astronomy and Space Science, University of Science and Technology of China, Hefei, 230026 China

## Abstract

As a new class of comet, main belt comets (MBCs) have attracted more and more attention in recent years. To study activity and physical properties of three MBCs 176P/LINEAR, 238P/Read and 288P/(300163) 2006 VW_139_, we carried out broadband CCD photometry of three MBCs on UT 2016 November 18–19 with the 1-m optical telescope at Lulin Observatory in Taiwan. By comparing cometary surface brightness profiles to stellar surface brightness profiles, and by comparing cometary absolute magnitude to the expected magnitude of inactive nucleus, we found that 176P/LINEAR was inactive, while 238P/Read and 288P/(300163) 2006 VW_139_ were active. By photometric studies, we obtained the *Afρ* values and the dust production rates. Finally, the activity of three MBCs were discussed. Our photometric results show that the total dust mass of 238P/Read and 288P/(300163) 2006 VW_139_ obtained in this work are of the same magnitude as the majority of known MBCs.

## Introduction

Comets are small bodies in solar system, they are distinguished from asteroids by the presence of coma or tail. The activity of comets are driven by water ice or sublimation of volatile admixtures. Before 2006, comets are believed to have formed in the outer solar system, beyond the orbit of Neptune, and to reside in two cold reservoirs: the Oort cloud and the Kuiper belt. Hsieh & Jewitt^1^ identified a third reservoir which is located at the main asteroid belt. Some asteroids show evidence for mass loss, these are called active asteroids. Active asteroids include main-belt comets (MBCs) and disrupted asteroids^2^. MBCs exhibit comet-like activity driven by the sublimation of volatile ice, while disrupted asteroids exhibit activity likely due to impacts^3–5^, rotational disruption^6–10^, thermal disintegration or electrostatics^11^.

MBCs have attracted most attention in recent years due to the implication from their activity that the existence of present-day ice in the asteroid belt. This offers opportunities to better understand the thermal and compositional history of our solar system, and place constraints on protosolar disk models. Research of MBCs may also be useful for investigating hypotheses that objects from the main asteroid belt may have played a significant role in the primordial delivery of water to the terrestrial planets^12–15^. Previous cometary measurements the deuterium-to-hydrogen (D/H) ratios in Jupiter family comets show that most of them are higher than the ocieanic D/H ratio and preclude the idea that the water on Earth is delilvered from Jupiter family^16^. Thus the possibility of the water on Earth is delilvered from the main asteroid belt has been enhanced.

There are 8 known unambiguous MBCs, which activity is driven by sublimation of volatiles and the triggering mechanism of activity of these objects are collision with a small impactor. (133P/Elst-Pizzaro, 176P/LINEAR, 238P/Read, 259P/Garradd, 324P/2010 R2 (La Sagra), 288P/(300163) 2006 VW_139_, P/2012 T1, 313P/Gibbs^17^. But not all of MBCs can reappear activity during the next perihelion passage, this may cast doubt on its sublimation-driven nature of the activity. To further determine whether main-belt objects are true MBCs, we need more observation data of perihelion passage. The objects of our observation are three MBCs 176P/LINEAR (118401), 238P/Read and 288P/(300163) 2006 VW_139_.

176P/LINEAR (hereafter 176P), also known as asteroid 118401, was discovered on September 7, 1999 by LINEAR telescope in Socorro, New Mexico. It is the third discovered member of the MBCs. It was discovered to exhibit cometary nature on 2005 November 26 by the Gemini North telescope on Mauna Kea in Hawaii^18^, but it was not exhibit activity during its 2011 perihelion passage, this casts doubt on the sublimation-driven nature of the activity observed in 2005^19^. The last perihelion passage of 176P was on 2017 March 12. Hsieh *et al*.^20^ examined the pole orientation and active region of 176P and suggested that the comet was active due to a seasonal variation of the solar flux at the active area.

238P/Read (formerly P/2005 U1, hereafter 238P) was discovered by M. T. Read using the Spacewatch 36 inch telescope on Kitt Peak on 2005 October 24. It was the second MBC to be discovered. When it was discovered, it showed cometary activity. 238P repeated activity during its 2011 and 2016 perihelion passage^21–23^. The last perihelion passage of 238P was on 2016 October 22.

288P/(300163) 2006 VW_139_ (also known as asteroid 300163, formerly 2006 VW_139_, hereafter 288P) was discovered in 2006 and first observed to be active on UT 2011 August 30^24^. 288P was reported the reactivation during its 2016 perihelion passage^23,25^. The last perihelion passage of 288P was on 2016 November 08. Agarwal *et al*.^26^ found that 288P is a binary main belt comet which ejected dust grains via ice sublimation and they suggested sublimation torques may play an important part in binary orbit evolution.

In this paper, we present optical observations and the surface brightness profile (SBP) of the above three MBCs observed on November 18–19, 2016. We also obtained the *Afρ* values and the dust mass production rates. The activity of three MBCs was discussed. Hsieh *et al*.^23^ published a paper about the 2016 reactivations of 238P and 288P recently, they reported observations of 238P and 288P from 2016 July to 2017 January in this paper. Our observation dates are in this time frame, but are not included in their observation Logs. Thus, the photometric results of our observations can be used to help fill in gaps of their observations.

## Methods

The three comets were observed by using the 1-m optical telescope at Lulin Observatory in Taiwan on 2016 November 18–19. This telescope has been equipped with an Alta U42 2 k × 2 k CCD camera. The pixel scale of camera is 0.348 arcsec, the field of view (FOV) is 11.9 × 11.9 arcmin^2^. The average seeing is 1.2 arcsec during the observations.

The three comets were observed through Asahi broad-band *R* filters. The effective wavelength of the *R* filter is *λ*_*e*_ = 6578 Å, the full width at halfmaximum (FWHM) is Δ*λ* = 1215 Å. The observation mode of telescope was set to track the sidereal motion, the exposure times of comets were chosen to make the apparent motion of the comet within the seeing disc. The details of observations are provided in Table 1.

**Table 1:** Log of all observations on UT 2016 November 18–19.

Comet	UT date	*R*_*h*_ (au)^a^	Δ(au)^b^	*α*(°)^c^	*ν*(°)^d^	*N*_*exp*_ × filter^e^	*t*_*exp*_ (s)^f^
176P/LINEAR	2016/11/19	2.633^*I*^	2.388	22.0	331.0	5 × *R*	150
238P/Read	2016/11/18	2.371^*O*^	1.615	18.7	3.1	5 × *R*	300
288P/(300163) 2006 VW_139_	2016/11/19	2.437^*O*^	1.954	22.7	8.0	5 × *R*	225

*Note*. ^a^The heliocentric distance in au, superscripts ‘*I*’ refers to the comet is inbound (pre-perihelion), ‘*O*’ refers to the comet is outbound (post-perihelion); ^b^The geocentric distance in au; ^c^The phase angle(Sun-comet-Earth) in degrees; ^d^The true anomaly in degrees; ^e^Number of exposures in the *R* filter; ^f^The total exposure time in second.

All images were reduced and calibrated in similar procedures (bias subtraction, flat-field correction and cosmic ray cleaning) used in our previous work^27^. The bias value used in the calibration was an average of several zero-exposure images. The final flat-fields were obtained from several images of the twilight sky. The night sky level used for photometry in the IRAF task PHOT was obtained from the region far from the nucleus. The NOMAD1 catalog was used to perform the magnitude calibration of the images. To minimize the effect of color terms, we selected Standard stars that optical colors were similar to the Sun.

## Results

### Cometary activity and surface brightness profile

**Figure 1:**
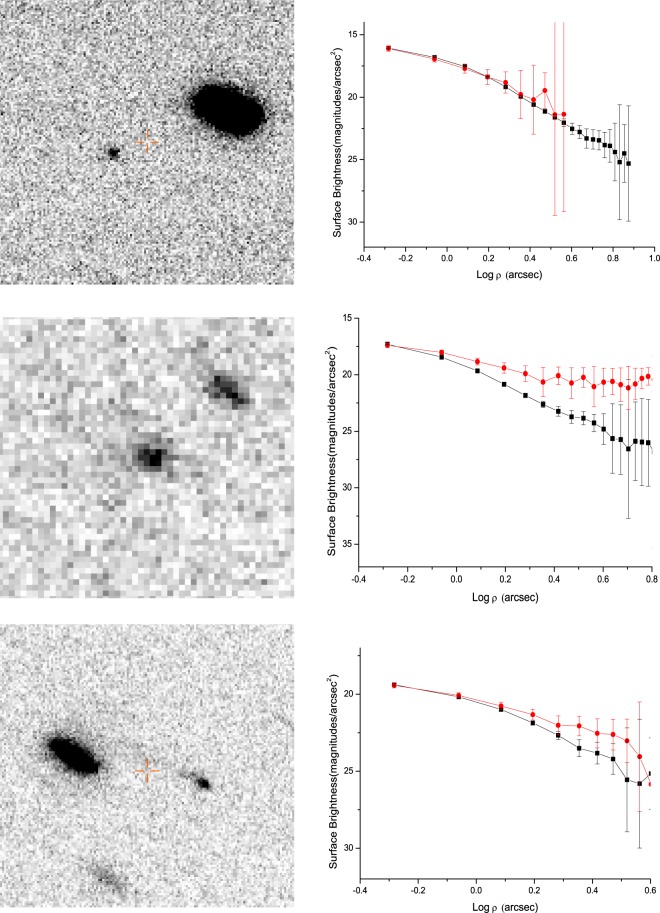
Co-added images (left-hand panels) and SBPs (right-hand panels) of 176P (top panels), 238P (middle panels) and 288P (bottom panels) in the *R* filter on November 19, 2016, November 18, 2016 and November 19, 2016, respectively. All images are oriented north-up (N), east-left (E). The field of view (FOV) of the top panel and bottom panel are 52.2 × 52.2 arcsec^2^, The field of view (FOV) of the middle panel is 17.4 × 17.4 arcsec^2^. The square dot line in SBPs represents the stellar SBP within the image, the circle dot line in SBPs represents the cometary SBP within the image.

All three MBCs looked like a stellar appearance in each single exposure frame. To increase the signal-to-noise ratios of both our target comets and field stars, we created two composite images per object, one combining all *R*-band images of each object aligned on the comet, and another combining all images of each object aligned on field stars. The combined frames still appear stellar (Fig. 1). To search possible the extent of coma, we extracted surface brightness profiles (SBPs) of comets and stars from the combined image using the method described in Shi & Ma^28^. By comparing with the stellar SBP, we find that 176P’s SBP is consistent with stellar SBP, 238P and 288P’s SBPs show a flux excess in outer region. This means that 176P was inactive or unresolved activity on November 19, 2016, while 238P and 288P were active on November 18, 2016 and November 19, 2016, respectively.

### The cometary dust production and dust mass production rate

The cometary dust production is usually made by means of the parameter *Afρ* value (cm)^29^. It is the product of the average grain albedo, the filling factor *f* (the ratio of the cross section of the dust grains to the field of view of aperture) and the projected radius of the photometric aperture *ρ*. *Afρ* can be given by the formalism

1 $$Af\rho =\frac{4{R}_{h}^{2}{{\rm{\Delta }}}^{2}{10}^{\mathrm{0.4(}{m}_{\odot }-{m}_{comet})}}{\rho },$$

where *R*_*h*_ is in AU, Δ and *ρ* are in cm, *m*_*comet*_ is the comet integrated magnitude. For a steady state coma, *Afρ* should be an aperture-independent parameter, this parameter can be used to compare measurements concerning the dust continuum produced under different observing conditions, times and instruments.

Theoretically, *Afρ* should be calculated using the magnitude of the coma. As the *Afρ* values obtained by other works were calculated by using the total magnitude, we used total magnitude *m*_*TOT*_ to calculate the *Afρ* values to facilitate comparison with other works. For 238P, *Afρ* value in the reference aperture of 1.8 arcsec is 5.08 ± 0.59 cm; for 288P, *Afρ* value in the reference aperture of 1.8 arcsec is 12.04 ± 1.11 cm (Table 2). The photometry apertures were computed by using the star’s FWHM in these coadded images that aligns the field stars.

**Table 2:** Magnitude, *Afρ*, dust mass production rate and total dust mass measured in *R*-band of comets 176P/LINEAR, 238P/Read and 288P/(300163) 2006 VW_139_

Comet	*m*_*TOT*_ (1.8 arcsec)^a^	*m*_*TOT*_ (4.0 arcsec)^b^	*Afρ* (cm)^c^	*Q*_*dust*_ (kg s^−1^)^d^	*M*_*dust*_ (10^7^kg)^e^
176P/LINEAR	20.01 ± 0.06	—	—	—	—
238P/Read	20.62 ± 0.05	20.06 ± 0.07	5.08 ± 0.59	1.9	2.8 ± 0.3
288P/(300163) 2006 VW_139_	19.95 ± 0.04	19.50 ± 0.06	12.04 ± 1.11	4.2	6.9 ± 1.5

*Note*. ^a^The total magnitude in the reference aperture of 1.8 arcsec; ^b^The total magnitude in the reference aperture of 4.0 arcsec; ^c^*Afρ* value in the reference aperture of 1.8 arcsec; ^d^The dust mass production rate in the reference aperture of 1.8 arcsec; ^e^The total dust mass in the reference aperture of 4.0 arcsec.

The dust mass loss rate can be calculated by dividing the total dust mass by the time of residence of the dust grains as they travel across the projected photometry annulus^30^. The coma magnitude between *ϕ*_1_ and *ϕ*_2_ is given by

2 $${m}_{d}=-\,2.5\,{\mathrm{log}}_{10}({10}^{-0.4{m}_{2}}-{10}^{-0.4{m}_{1}}),$$

where *m*_1_ and *m*_2_ are the magnitudes in apertures of *ϕ*_1_ and *ϕ*_2_. To minimize the effects of nucleus contribution and sky background, we choose *ϕ*_1_ = 2.4 arcsec and *ϕ*_2_ = 3.0 arcsec. The dust mass is given by Jewitt^30^ as ${M}_{d}=\frac{4}{3}\sigma {a}_{dust}{C}_{d}$, where *σ* is bulk density, *a*_*dust*_ is average grain radii, *C*_*d*_ is the total cross-section of the coma dust particles and can be computed from Equation (1). The time of residence of the dust grains in the annulus between *ϕ*_1_ and *ϕ*_2_ is *τ*(*R*_*h*_) = 1.5 × 10^11^Δ(*ϕ*_2_ − *ϕ*_1_)/*v*_*ej*_^30^, where *τ*(*R*_*h*_) is in s, Δ is in AU, *ϕ*_1_ and *ϕ*_2_ are expressed in radians,*v*_*ej*_ is the radial outflow speed of the dust grains from the nucleus in m s^−1^. For comet Hale-Bopp, expansion measurements showed that the radial outflow speed of gas from the nucleus was ${v}_{{R}_{h}}={v}_{0}{({r}_{0}/{R}_{h})}^{1/4}$ ^31^, where *v*_0_ = 550 ms^−1^ and *r*_0_ = 5 au. For spherical grains emitted from a homogeneous nucleus, a dust velocity is about 10 per cent of the gas velocity^32,33^. So the dust grain ejection velocity is *v*_*ej*_ = *v*(*R*_*h*_)/10. We adopted the dust grain radius value of *a*_*dust*_ = 10 *μ*m, this value consistents with dust modeling results for 133P^34^. The grain density was adpoted the canonical asteroid density *σ* = 2000 kg m^−3^ ^35^. For 238P, *m*_1_ = 20.47 ± 0.06, *m*_2_ = 20.29 ± 0.06, the calculated dust production rate is 1.9 kg s^−1^. For 288P, *m*_1_ = 19.75 ± 0.05, *m*_2_ = 19.61 ± 0.05, the calculated dust production rate is 4.2 kg s^−1^ (Table 2).

## Discussion

For 176P, Hsieh *et al*.^20^ derived best-fit IAU phase function parameters of *H* = 15.10 ± 0.05 and *G* = 0.15 ± 0.10. Using these phase function parameters, Hsieh *et al*.^19^ summarized apparent *R*-band magnitude and absolute *R*-band magnitude of 176P observed before 2014. To compare previous observation to ours, we computed absolute magnitudes based on the total magnitude using the *HG* approximation with scattering parameter *G* = 0.15 ± 0.1 and obtained *m*_*R*_(1, 1, 0) = 14.96 ± 0.22. Considering rotational variations of 176P is about 0.7 mag (a peak-to-trough photometric range)^20^. The absolute magnitude obtained in this work is still in the range of rotational variations, this also suggest that it was inactive on November 19, 2016.

Table 3 summarized available *R*-band photometry results of comet 238P. The inactive photometric behavior of 238P has been previously established by Hsieh, Meech & Pittichová^21^ who derived best-fit IAU phase function parameters of *H* = 19.05 ± 0.05 mag and *G* = −0.03 ± 0.05. Using *G* = −0.03 ± 0.05, we can then compute the equivalent absolute magnitudes (at heliocentric and geocentric distances of *R*_*h*_ = Δ = 1 au and a solar phase angle of *α* = 0°) for all observations of 238P (Table 3). Comparing absolute magnitude and *Afρ* values obtained in this work to previous observation, we can find that there is an obvious about 2 mag photometric enhancement in this work than data obtained in 2010 July and August when 238P was observed to be largely inactive, this also suggest that it was active on November 18, 2016. Table 4 summarized available *R*-band photometry results of comet 288P. Absolute *R*-band magnitudes (at *R*_*h*_ = Δ = 1 au and *α* = 0°), were computed by using *G* = 0.15 ± 0.1^20^ (Table 4).

**Table 3:** Summary of available *R*-band photometry results of comet 238P/Read.

Active?^a^	UTdate	*R*_*h*_ (au)^b^	Δ(au)^c^	*α*(°)^d^	*ν*(°)^e^	*m*_*TOT*_^f^	*m*(1, 1, 0)^g^	*Afρ* (cm)^h^	References
Perihelion	2005-07-28	2.365	2.276	25.2	0.0	—	—	—	—
yes	2005-11-10	2.436	1.446	0.6	31.4	19.28 ± 0.05	16.41 ± 0.17	7.47 ± 0.86	^39^
yes	2005-11-19	2.448	1.468	3.8	33.9	19.34 ± 0.05	16.13 ± 0.16	7.23 ± 0.83	^39^
yes	2005-11-20	2.450	1.471	4.3	34.2	19.46 ± 0.05	16.20 ± 0.15	6.47 ± 0.74	^39^
yes	2005-11-21	2.451	1.475	4.8	34.5	19.37 ± 0.05	16.07 ± 0.15	7.08 ± 0.82	^39^
yes	2005-11-22	2.453	1.480	5.3	34.8	19.28 ± 0.05	15.94 ± 0.15	7.69 ± 0.89	^39^
yes	2005-11-26	2.459	1.499	7.1	35.9	19.72 ± 0.10	16.24 ± 0.17	5.24 ± 1.21	^39^
yes	2005-12-24	2.504	1.739	17.1	43.6	20.12 ± 0.03	15.79 ± 0.12	4.34 ± 0.30	^39^
yes	2005-12-25	2.505	1.751	17.4	43.9	20.16 ± 0.03	15.80 ± 0.12	4.24 ± 0.29	^39^
no	2007-01-27	3.433	2.488	5.2	123.0	24.90 ± 0.40	19.71 ± 0.42	0.14 ± 0.13	^39^
Aphelion	2008-05-19	3.963	3.276	11.8	180.0	—	—	—	—
no	2010-07-07	2.704	1.821	13.0	−68.2	23.61 ± 0.10	19.19 ± 0.16	0.21 ± 0.05	^21^
no	2010-07-20	2.674	1.709	8.5	−65.2	22.85 ± 0.06	18.82 ± 0.15	0.39 ± 0.05	^21^
no	2010-08-15	2.616	1.608	2.6	−58.9	22.34 ± 0.05	18.88 ± 0.16	0.57 ± 0.07	^21^
no	2010-09-03	2.576	1.643	10.7	−54.1	21.97 ± 0.04	17.99 ± 0.13	0.79 ± 0.07	^21^
yes	2010-09-04	2.574	1.647	11.0	−53.9	22.01 ± 0.05	18.02 ± 0.14	0.76 ± 0.09	^21^
yes	2010-09-05	2.572	1.651	11.4	−53.6	22.02 ± 0.05	18.00 ± 0.14	0.76 ± 0.09	^21^
yes	2010-10-05	2.514	1.869	20.3	−45.7	22.25 ± 0.05	17.62 ± 0.12	0.66 ± 0.08	^21^
yes	2010-11-25	2.433	2.414	23.5	−31.5	21.75 ± 0.05	16.51 ± 0.11	1.27 ± 0.15	^21^
yes	2010-12-09	2.416	2.566	22.5	−27.5	21.86 ± 0.07	16.54 ± 0.12	1.20 ± 0.19	^21^
Perihelion	2011-03-10	2.361	3.277	7.9	0.0	—	—	—	—
Perihelion	2016-10-22	2.366	1.410	8.7	0.0	—	—	—	—
yes	2016-11-18	2.371	1.615	18.7	3.1	20.62 ± 0.05	16.50 ± 0.12	5.08 ± 0.59	This work

*Note*. ^a^Is visible activity detected?; ^b^The heliocentric distance in au; ^c^The geocentric distance in au; ^d^The phase angle (Sun-comet-Earth) in degrees; ^e^The true anomaly in degrees; ^f^The total magnitude; ^g^Absolute *R*-band magnitude; ^g^*Afρ* values in *R*-band.

**Table 4:** Summary of available *R*-band photometry results of comet 288P/300163.

Active?^a^	UTdate	*R*_*h*_ (au)^b^	Δ(au)^c^	*α*(°)^d^	*ν*(°)^e^	*m*_*TOT*_^f^	*m* (1, 1, 0)^g^	References
Perihelion	2011-07-18	2.438	2.293	24.6	0.0	—	—	—
yes	2011-11-14	2.506	1.561	8.4	33.2	18.62 ± 0.05	15.08 ± 0.27	^24^
yes	2011-11-14	2.506	1.561	8.4	33.2	18.64 ± 0.05	15.10 ± 0.27	^24^
yes	2011-11-18	2.510	1.586	10.0	34.3	18.60 ± 0.10	14.95 ± 0.28	^24^
yes	2011-11-19	2.512	1.596	10.6	34.6	18.64 ± 0.10	14.95 ± 0.27	^24^
yes	2011-11-30	2.525	1.685	14.4	37.4	19.04 ± 0.05	15.08 ± 0.24	^24^
yes	2011-12-04	2.530	1.724	15.6	38.5	19.12 ± 0.03	15.07 ± 0.23	^24^
yes	2011-12-19	2.549	1.895	19.2	42.4	19.68 ± 0.03	15.29 ± 0.22	^24^
yes	2012-01-07	2.577	2.152	21.7	47.4	20.43 ± 0.10	15.66 ± 0.23	^24^
yes	2012-10-14	3.111	3.273	17.7	107.1	22.45 ± 0.03	16.49 ± 0.23	^36^
Aphelion	2014-03-13	3.660	2.697	4.5	180.0	—	—	—
Perihelion	2016-11-08	2.436	1.823	21.3	0.0	—	—	—
yes	2016-11-19	2.437	1.954	22.7	8.0	19.95 ± 0.04	15.48 ± 0.21	This work

*Note*. ^a^Is visible activity detected?; ^b^The heliocentric distance in au; ^c^The geocentric distance in au; ^d^The phase angle(Sun-comet-Earth) in degrees; ^e^The true anomaly in degrees; ^f^The total magnitude; ^g^Absolute *R*-band magnitude.

As the determination of the dust production rate of a comet is highly model-dependent and parameter-dependent, we can’t compare the dust production rates obtained in this work to other works directly. To compare activitiy of 238P and 288P in this work to Hsieh *et al*.’s^23^ work and other MBCs. We computed the total mass *M*_*dust*_ of visible ejected dust by using of equation^23^

3 $${M}_{dust}=\frac{4}{3}\pi {a}_{N}^{2}{a}_{dust}{\rho }_{d}\frac{1-{10}^{0.4(m(1,1,0)-H)}}{{10}^{0.4(m(1,1,0)-H)}},$$

where *a*_*N*_ is the nucleus’s radius in m. For consistency, we choose dust grain densities of *ρ*_*d*_ = 2500 kg s^−1^, mean effective grain radii of *a*_*dust*_ = 1 mm and the photometry aperture radius is 4 arcsec, these values are same with Hsieh *et al*.’s work^23^. For 238P, the total apparent *R*-band magnitude measured in aperture radius 4 arcsec is *m*_*TOT*_ = 20.06 ± 0.07, the total absolute *R*-band magnitude computed by using *H* = 19.05 ± 0.05 mag and *G* = −0.03 ± 0.05^21^ is *m*(1, 1, 0) = 15.94 ± 0.13, *a*_*N*_ ≈ 0.4 km^21^, the corresponding total dust mass is *M*_*dust*_ = (2.8 ± 0.3) × 10^7^ when 238P was at true anomaly *ν* = 3°.1 on 2016 November 18. For 288P, the total apparent *R*-band magnitude measured in aperture radius 4 arcsec is *m*_*TOT*_ = 19.50 ± 0.06, the total absolute *R*-band magnitude computed by using *H* = 16.80 ± 0.12 and *G* = 0.18 ± 0.11^23^ is *m*(1, 1, 0) = 15.07 ± 0.23, *a*_*N*_ ≈ 1.3 km^36^, the corresponding total dust mass is *M*_*dust*_ = (6.9 ± 1.5) × 10^7^, when 288P was at true anomaly *ν* = 8°.0 on 2016 November 19. Hsieh *et al*.^23^ reported 238P’s total dust mass of *M*_*dust*_ = (2.3 ± 0.3) × 10^7^ on 2016 November 5 (when 238P was at *ν* = 4°.2 and 288P’s total dust mass of *M*_*dust*_ = (6.8 ± 1.4) × 10^7^ on 2016 November 28 (when 288P was at *ν* = 5°.6 Examining previously reported photometry of active dust emission, Hsieh *et al*.^23^ found that activity of 238P in 2016 were lower than the activity in 2010 and the activity of 288P in 2016 were larger than the activity in 2000. Comparing the total dust mass of 238P and 288P obtained in this work to Hsieh *et al*.’s^23^ work, we find that our results are consistent with Hsieh *et al*.’s^23^ conclusions. Comparing the total dust mass of 238P and 288P obtained in this work to other MBCs^37,38^, we find that the total dust mass of 238P and 288P obtained in this work are of the same magnitude as the majority of known MBCs’s. This is consistent with the fact that almost all of the MBCs appear to eject nearly identical quantities of dust^37^.

## Data Availability

Observations were carried out with the 1-m optical telescope at Lulin Observatory in Taiwan. The observation data can be obtained from Lulin Observatory.
